# Antigen-Specific Tolerogenic Dendritic Cells Ameliorate the Severity of Murine Collagen-Induced Arthritis

**DOI:** 10.1371/journal.pone.0131152

**Published:** 2015-06-24

**Authors:** Bin Ning, Jianlu Wei, Aijun Zhang, Weiming Gong, Jinqiu Fu, Tanghong Jia, Shang-You Yang

**Affiliations:** 1 Department of Orthopedic Surgery, Jinan Central Hospital affiliated with Shandong University, Jinan, Shandong, China; 2 Department of Pediatrics, Qilu Hospital, Shandong University, Jinan, Shandong, China; 3 Department of Biological Sciences, Wichita State University, Wichita, KS, United States of America; Department of Immunology, CHINA

## Abstract

Dendritic cells (DCs) play important roles in initiation of the pathogenic processes of autoimmune disorders, such as rheumatoid arthritis (RA). Tolerogenic dendritic cells (tolDCs) are generated from naïve DCs and induce T cell tolerance; thus, they represent a promising strategy for specific cellular therapy for autoimmune diseases. In this study, we generated green fluorescent protein (GFP)-labeled tolDCs and confirmed their phenotypes and biological functions. We found that tolDCs suppressed the memory lymphocyte response and exhibited strong tolerogenic potential; thus, these cells show promise for the treatment of autoimmune diseases. Additionally, a collagen-induced arthritis (CIA) mouse model was used to test the role of tolDCs *in vivo*. The results of a further mechanistic experiment revealed that tolDCs suppressed inflammatory arthritis at least partially by up-regulating regulatory T (Treg) cells. Collectively, our data suggest that tolDCs may be used as a promising alternative therapy for inflammatory arthritis.

## Introduction

Rheumatoid arthritis (RA) is a common autoimmune disease characterized by synovitis and progressive cartilage and subchondral bone destruction [1]. The current treatment regimens for RA include immunosuppressive drugs and biological agents [2]. However, these therapeutic agents can induce generalized immune suppression, which can increase the risk of infectious diseases [3]. Therefore, new therapeutic approaches are needed to control disease progression without compromising the immune system of the patient [4]. Many types of immune cells are involved in the pathogenesis and progression of RA, including dendritic cells (DCs), monocytes and macrophages, T and B lymphocytes, neutrophils and natural killer (NK) cells [5]. DCs are professional antigen-presenting cells (APCs) that have the capacity to stimulate or to inhibit immune responses [6]. DCs that directly interact with antigen-specific T cells and induce autoimmune tolerance are called tolerogenic dendritic cells (tolDCs), which have great potential for the prevention and treatment of autoimmune diseases. It is well known that the innate immune system down-regulates effector mechanisms and restores homoeostasis in injured tissues via IL-10 and transforming growth factor (TGF) family cytokines, which have the capacity to induce regulatory T cells activity, inhibit production of pro-inflammatory cytokines and promote tissue healing by regulating extracellular matrix protein deposition and angiogenesis. These cytokines have been clinically tested in patients with inflammatory bowel disease, RA, autoinflammatory syndromes, fibrosing processes and malignancy[7]. Cellular therapy based on tolDCs is a novel and promising means to efficiently eradicate clinical symptoms and ameliorate immune responses in autoimmune patients[8–11]. Furthermore, DCs exposed to immunosuppressive cytokines, such as IL-10 and TGF-β, have been reported to have strong tolerogenic potentials [12]. Indeed, our previous study has identified tolerogenic characteristics of IL-10-induced DCs [13]. Other studies have also reported that tolDC-induced tolerance leads to the effective suppression of experimental autoimmune encephalomyelitis [7] and pulmonary inflammation [8–11]. Indeed, tolDCs represent a promising immunosuppressive therapeutic tool with which to attenuate pathogenic T cell responses in autoimmune arthritis [14].

Collagen-induced arthritis (CIA) is a well-established mouse model of experimental arthritis with many of the pathological characteristics of human RA [15]. We hypothesized that type II collagen (CII)–pulsed tolDCs would induce antigen-specific immune tolerance in autoimmune disorders (AIDs) and would ameliorate arthritis progression. In this study, we describe a line of green fluorescent protein (GFP)-transduced mouse bone marrow-derived collagen II-specific tolDCs (CII-tolDCs) that exhibited dramatic therapeutic effects in a mouse CIA model. Furthermore, we found that these therapeutic effects were due to up-regulation of the Treg population. These antigen-specific tolDCs may represent a new avenue of research for the development of future clinical treatments that do not disturb the normal immune system.

## Materials and Methods

### *In vitro* experiments

#### Generation and cultivation of tolDCs and GFP-tolDCs

The isolation of bone marrow-derived dendritic cells (BmDCs) was performed in the presence of granulocyte-macrophage colony stimulating factor (GM-CSF) (10 ng/ml) and IL-4 (1 ng/ml) using established protocols [16]. Mature dendritic cells (mDCs) were isolated by stimulation with lipopolysaccharides (LPS; 1 μg/ml) for 2 days. tolDCs were obtained by culturing BmDCs with IL-10 (20 ng/ml) and TGF-β (20 ng/ml) for 6 days. Then, the tolDCs were purified to approximately 95% purity using anti-CD11c microbeads. The phenotypes and functional activities of mDCs and tolDCs were determined according to the protocols described in the following sections.

The purified mDCs or tolDCs at 5×10^5^ cells/mL were co-cultured with recombinant lentiviral vectors encoding the GFP gene (Lenti-GFP, MOI = 10, ANNGEN, Beijing, China), as detailed previously [17]. CII at a final concentration of 1μg/ml was added to the cultures on the next day, and the cells were harvested on day 4.

#### Phenotype identification

tolDCs were stained with FITC-labeled CD11c, MHC-II monoclonal antibody (mAb) and PE-conjugated CD80 or CD86 antibody (Biolegend, CA, USA), and they were then analyzed by flow cytometry using a FACScan flow cytometer and CellQuest software (Becton Dickinson, Mountain View, CA, USA). CD11-positive signals indicated the DC phenotype. MHC-II functions as the first stimulatory signal in the antigen-presenting process, while CD80 and CD86 are costimulatory molecules that promote T cell activation. The expression levels of CD80, CD86, and MHC-II should be decreased in tolDCs. The data are expressed as the mean fluorescence intensity (MFI).

#### RT-PCR

Total RNA was extracted from the articular cartilage of the CIA mice or from cultured chondrocytes using an RNeasy kit (Qiagen Inc., Valencia, CA, USA), and first-strand cDNA was generated using an ImProm-II reverse transcription system (Promega, Madison, WI, USA). Real-time PCR was performed using SYBR Green I dye to amplify the target genes. Primer pairs for each target gene were designed to generate products of between 100 bp and 200 bp in length. The sequences of the specific primers used to amplify the mouse genes were as follows: IL-12: 5’-CGGCAGCAGAATAAATATGAGAACT-3’ and 5’-GGCGGGTCTGGTTTGATG-3’; CCR7: 5’-CATTGCCTATGACGTCACCTACA-3’ and 5’-GAAGGCATACAAGAAAGGGTTGA-3’; FoxP3: 5’-CACTGCCCCTAGTCATGGT-3’ and 5’-GGAGGAGTGCCTGTAAGTGG-3’; STAT-5A: 5’-GACCCGCCAACAAATTAAGA-3’ and 5’-TCGTGGTAAACTGGACACCA-3’; STAT-5B: 5’-GTGAAGCGCTCAACATGAAA-3’ and 5’-ACTGGGACCAGGACACAGAC-3’; IL-10: 5’-ACAGCCGGGAAGACAATAAC-3’ and 5’-CAGCTGGTCCTTTGTTTGAA-3’; and GAPDH: 5’-AGAACATCATCCCTGCATCC-3’ and 5’-AGTTGCTGTTGAAGTCGC-3’.

#### Mixed leukocyte reaction

Splenic leukocytes isolated from DBJ/1 mice were co-cultured in 96-well plates for 4 days in the presence of equal numbers of DCs and tolDCs (and 25 μg/ml mitomycin) at a final volume of 200 μl per well (Costar, Cambridge, MA, USA). To determine the optimal cell interaction capacity, sequential cell densities of 10^4^, 10^5^, 10^6^, and 10^7^/ml were used. A WST-8 assay kit (Beyotime Institute of Biotechnology, Haimen, China) was used to estimate lymphocyte proliferation for each group as previously described [17].

### *In vivo* experiments and assessments

#### Mice

The institutional ethical committee approved all of the animal procedures. Male DBA/1Lac/J mice (7–8 weeks of age) were purchased from the Vital River Laboratory Animal Technology Co. (Beijing, China). The mice were maintained for 2 weeks prior to experimentation in a specific pathogen-free (SPF) animal facility at the Experimental Animal Center of Jinan Central Hospital affiliated with Shandong University.

#### Induction of arthritis

Chicken CII (Chondrex LLC, Redmond, WA, USA) was dissolved in 0.05 M acetic acid to a concentration of 2 mg/ml and was then emulsified in an equal volume of complete Freund’s adjuvant (CFA) by stirring overnight at 4°C. The DBA/1Lac/J mice were intradermally immunized at 1.5 cm from the base of the tail with 50 μl (50 μg of CII) of the emulsion. On day 21 after the first immunization, the mice received booster injections containing 50 μg of CII emulsified in incomplete Freund’s adjuvant (IFA).

#### Clinical CIA scoring and mouse grouping

The arthritic mice were monitored every other day to record arthritis progression in the paws as previously described [18]. Each paw was assigned a clinical score according to the following scale:

1. No evidence of erythema or swelling;
2. Erythema and mild swelling confined to the tarsals or ankle joint;
3. Erythema and mild swelling extending from the ankle to the tarsals;
4. Erythema and moderate swelling extending from the ankle to the metatarsal joints; or
5. Erythema and severe swelling encompassing the ankle, foot and digits or ankylosis of the limb.

The maximum cumulative score for each mouse was 16. On the day that the score reached 7 (moderate disease severity), the mouse was assigned to one of the following three groups: the “tolDC group” (Group A, n = 12), which received 1x10^6^ tolDCs by intravenous injection (i.v.); the “mDC group” (Group B, n = 12), which was administered 1x10^6^ mDCs by i.v. transfusion; and the “Control group” (Group C, n = 12), which received 1 ml saline injection. The mice were monitored, and the clinical scores were recorded for 6 weeks.

#### Histological examination

Six weeks after treatment, the animals were sacrificed by CO_2_ narcosis plus cervical dislocation. Their arthritic paws, spleens and livers were harvested. Each paw was divided into two parts along the median longitudinal axis. One half was fixed in 10% formalin for histological examination, and the other half was snap frozen for plastic sectioning.

Briefly, the joint tissues were fixed in cold paraformaldehyde overnight and dehydrated in a gradient series of alcohol followed by Jaffaite embedding and light polymerization. The plastic-embedded joint tissue was machine-chipped (EXAKT 300CP) to create 100 μm sections and (EXAKT400CS/AW) polished to a 30 μm thickness. Then, the sections were subjected to routine histological processing and hematoxylin and eosin (H&E) staining. Histological evaluation of the arthritic paws of the animals from the three groups was conducted in a blinded fashion. For each paw section, the extent of synovitis, pannus formation, and bone/cartilage destruction was determined using an established grading system as follows [19]: grade 0—no signs of inflammation; grade 1—mild inflammation with hyperplasia of the synovial lining; grade 2—pannus formation and superficial cartilage erosion; grade 3—major erosion of the cartilage and subchondral bone; and grade 4—architectural changes and destruction.

The spleen and liver tissues were paraffin-embedded after fixation, cut into 5 μm-thick sections, mounted on glass slides and stained with H&E. For the tolDC trafficking study, portions of the spleen and liver tissues were fixed in a 4% paraformaldehyde solution overnight and then snap frozen in embedding medium (10% polyvinyl alcohol and 4% polyethylene glycol). Next, the specimens were cut into 5 μm-thick sections using a cryostat and observed by fluorescence microscopy (BX53, Olympus, Japan).

#### Enzyme-linked immunosorbent assay (ELISA)

Production of cytokines, such as IFN-γ, IL-10, and TGF-β, in murine sera or *in vitro* cell culture media was quantified by ELISA (ANNGEN, China) according to the manufacturer’s protocol. The serum levels of CII antibody isotypes (IgG1, IgG2a, IgG2b, and IgG3) were also determined by ELISA. Briefly, a 96-well Immuno-Maxisorp Plate (Nunc, Roskilde, Denmark) was coated with murine CII (10 mg/ml) or the tested cytokines (IFN-γ, IL-10, or TGF-β, 2 mg/ml) overnight at 4°C and was then blocked with 10% FBS suspended in PBS. Serum samples were diluted to 0.1% (vol:vol) and were incubated for 2 h at 37°C. After washing, bound antibody isotypes were detected using biotin-conjugated anti-rat whole IgG (heavy and light chain) or IgG1, IgG2a, IgG2b, and IgG3 antibodies (Pharmingen, San Diego, CA, USA). Then, the plates were washed and incubated with 100 ml of 1 mg/ml 2,2’-azino-di-(3-ethylbenzthiazoline sulfonate) substrate (ABTS; Boehringer Mannheim, Indianapolis, IN), and absorbance was measured at 405 nm [20].

#### Flow cytometry

CD4+CD25+T cells were isolated from mouse spleens and were enriched using a CD4+CD25+T Cell Isolation Kit with a MidiMACS Separator according to the manufacturer’s instructions (Miltenyi, Bergisch Gladbach, Germany). For intracellular Foxp3 staining, cells were incubated with Cy-chrome-labeled anti-CD4 and FITC-labeled anti-CD25 mAbs, and they were then fixed and stained with an anti-mouse Foxp3 mAb, according to the instructions in the operating manual (eBioscience, San Diego, CA, USA).

#### Soft X-ray assay

X-ray radiographic analysis was performed to examine CIA mouse paws. Bone morphology (analyzed at 5.0 kV for 6.0 s; Faxitron X-ray, Wheeling, IL) was examined at 7 weeks after the first immunization.

#### MicroCT assay

Prior to histological processing, paraformaldehyde-fixed samples were evaluated by microCT using a Scanco vivaCT40 cone beam scanner (SCANCO Medical, Switzerland) with a 55kVp source and 14 5mA current. We scanned the samples at a resolution of 10.5 mm.

#### Statistical analyses

All of the *in vitro* experiments were repeated 3 times to ensure data reproducibility. Statistical analysis was performed using SPSS statistical package (SPSS, Chicago, IL, USA), version 11.0. All of the data are presented as the mean ± SD. Differences between two groups were compared using the independent samples t-test, whereas multiple groups were compared by one-way analysis of variance (ANOVA), and the LSD test was used for post hoc multiple comparisons. A *p*-value of <0.05 was considered statistically significant.

## Results

### tolDCs retained their tolerogenic phenotype and function

**Fig 1:**
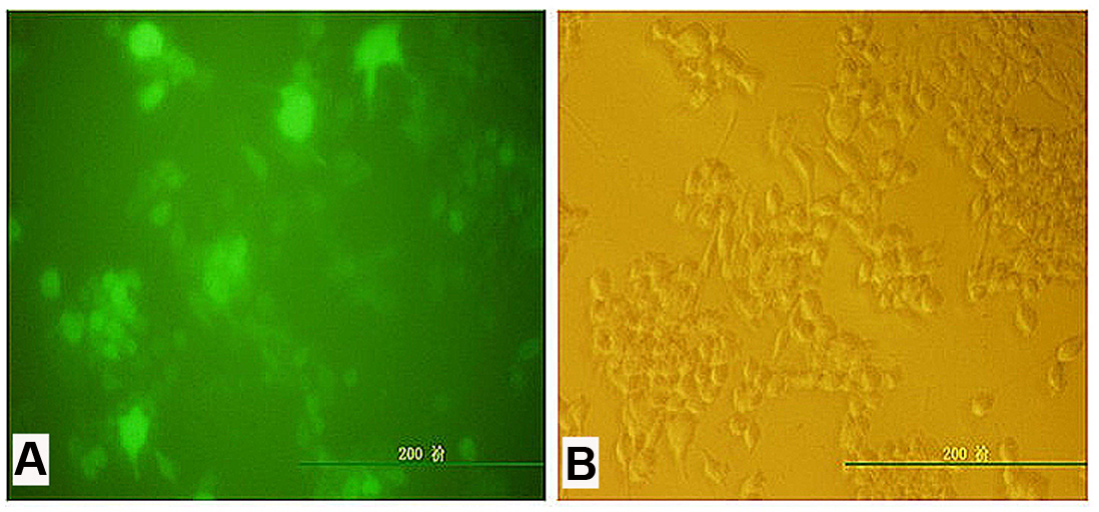
GFP-transduced tolDCs imaged by fluorescence (A) and bright-field microscopy (B). The transduction efficiency was approximately 95% based on an MOI of 10.

The GFP gene was successfully transduced into both mDCs and tolDCs with lentiviruses, as shown in Fig 1. Transgene expression was observed as bright green fluorescent emission by fluorescence microscopy, and the transduction efficiency was greater than 95%. The GFP-labeled mDCs and tolDCs were stored at -80°C for future CIA mouse model treatments.

**Fig 2:**
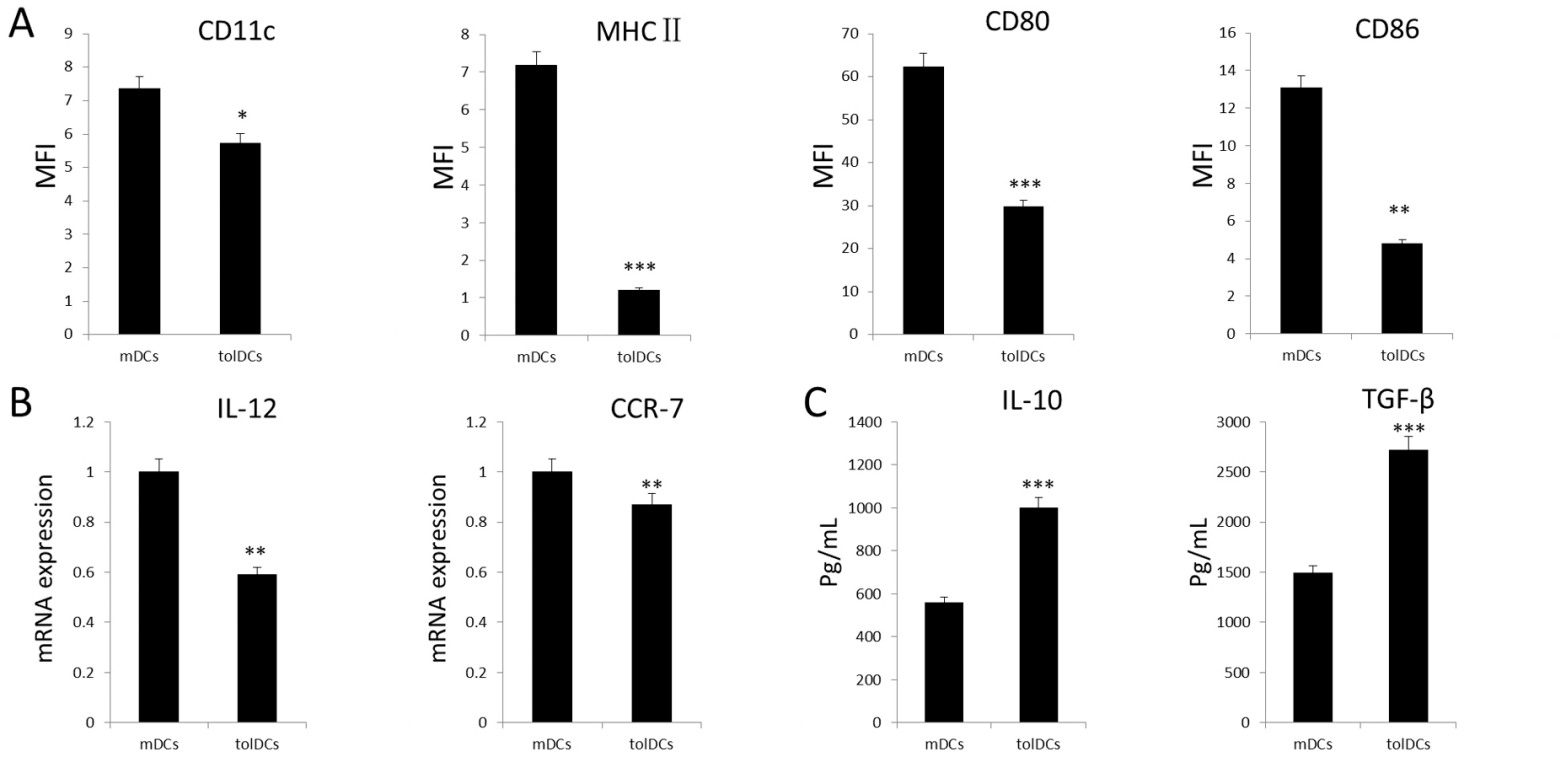
tolDC tolerogenic phenotype and function. (A) Flow cytometry analysis to quantify the expression levels of CD11c, MHC-II, CD80, and CD86, which are cell markers for mDCs and tolDCs; (B) IL-12 and CCR7 mRNA expression levels (mean ± SD from 3 cases) in dendritic cells, as determined by real-time PCR; and (C) IL-10 and TGF-β protein levels in DC culture media on day 6 of the experiment (n = 6). *p<0.05, **p<0.01, ***p<0.001.

To confirm the phenotype and function of the GFP-labeled tolDCs, flow cytometry was employed to identify the cell surface expression of CD11c, MHC-II, CD80, and CD86 on mDCs and tolDCs. As illustrated in Fig 2A, the expression of these makers was significantly lower on tolDCs compared with that on mDCs. To further confirm the tolerogenic function of tolDCs, real-time PCR was conducted, revealing that the expression levels of the pro-inflammatory cytokine IL-12 and chemokine receptor CCR7 were significantly decreased in tolDCs compared with mDCs (Fig 2B). In addition, the IL-10 and TGF-β protein levels in the culture medium containing tolDCs were up to 2- to 3-fold higher than those in the culture medium containing mDCs. Collectively, the tolDCs exhibited a distinct tolerogenic phenotype and immune-suppressive function.

### tolDCs showed decreased immunogenic response *in vitro*

**Fig 3:**
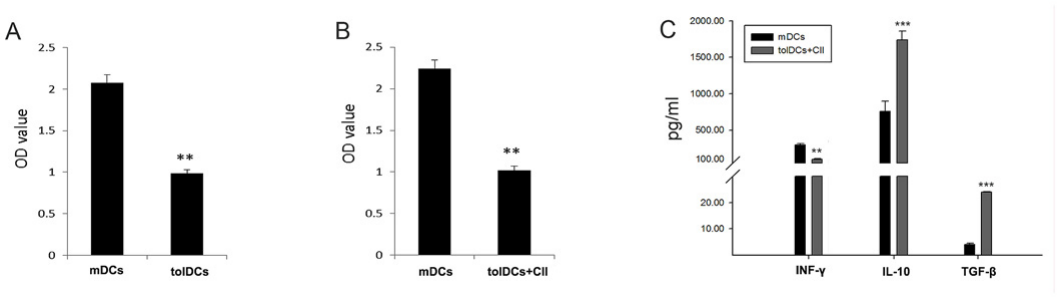
tolDCs inhibit lymphocyte proliferation and immune response. (A) tolDCs dramatically inhibited lymphocyte proliferation compared with mDCs. (B) CII-specific tolDCs showed a stronger ability to inhibit lymphocyte proliferation than mDCs. (C) CII-specific tolDCs dramatically induced the expression of the anti-inflammatory cytokines IL-10 and TGF-β and decreased the expression of the pro-inflammatory cytokine INF-γ. *p<0.05, **p<0.01, ***p<0.001.

To further investigate the immune response ability of tolDCs, we performed co-culture assay with primary splenocytes. The proliferation capacity of mouse splenic leukocytes following co-culture with mDCs or tolDCs was determined by CCK-8 assay. Fig 3A indicates that the tolDCs had a weaker influence on lymphocyte proliferation than the mDCs (*p*<0.01).

To further determine whether CII-tolDCs promoted the tolerance of memory lymphocytes, splenic lymphocytes were isolated from CIA mice and co-cultured with tolDCs or mDCs. Whereas mDCs robustly promoted CIA splenic leukocyte proliferation, CII-tolDCs showed a decreased capacity for promoting leukocyte proliferation (Fig 3B, *p*<0.01). With regard to cytokine production, CII-tolDCs promoted a decrease in the IFN-γ level and increases in the IL-10 and TGF-β levels compared with mDCs (Fig 3C).

### tolDCs ameliorated inflammatory arthritis *in vivo*

**Fig 4:**
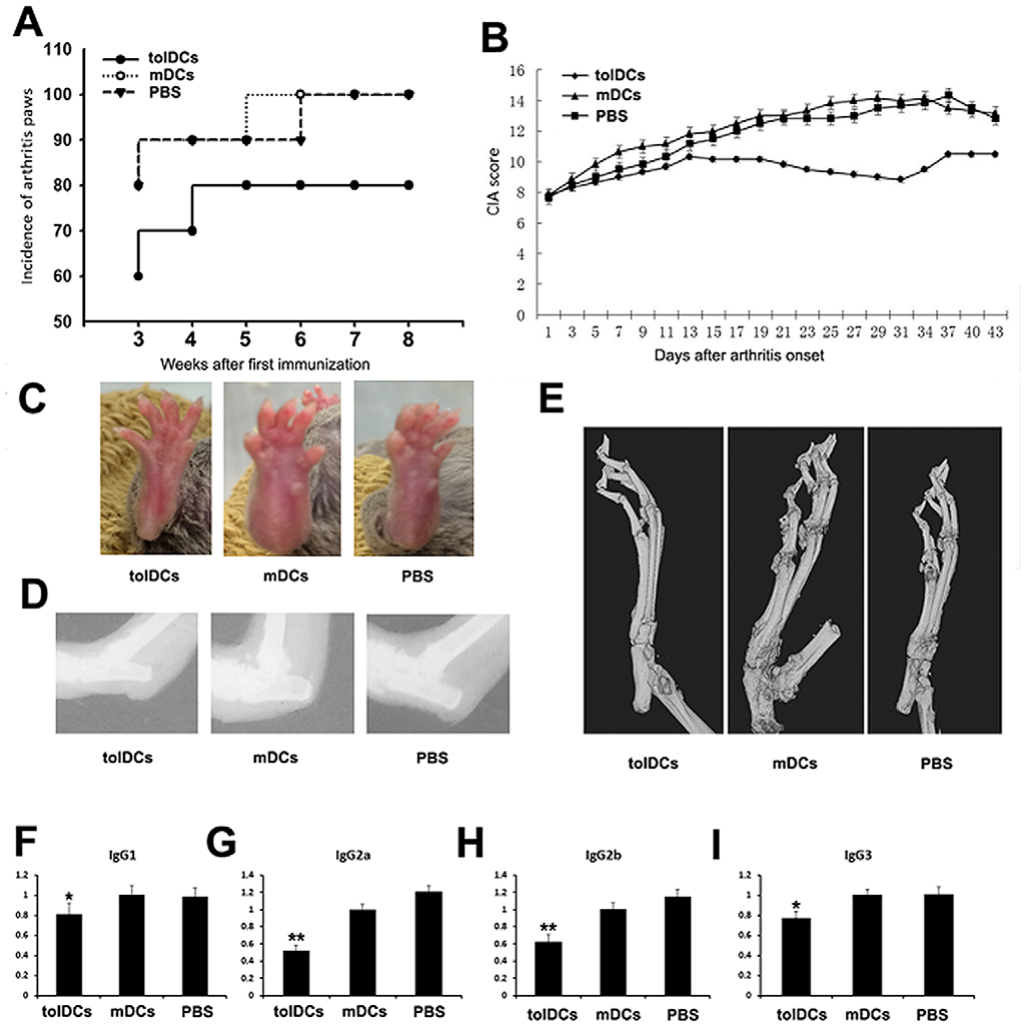
tolDCs prevent the development of inflammatory arthritis. (A) The incidence of arthritic paws from week 3 and after the first immunization. (B) Clinical scores of the CIA mice, beginning on the first day of cell transfusion. (C) Phenotypes of the CIA-induced mice in the tolDC-, mDC- and PBS-treated groups, respectively. (D-E) The results of X-ray and microCT analyses for each group. (F-I) Isotypes of CII-specific IgG1, IgG2a, IgG2b and IgG3 in sera from 5 mice from each group. *p<0.05, **p<0.01.

To further determine whether tolDCs have therapeutic effects on inflammatory arthritis, we administered purified tolDCs and mDCs to CIA mice and observed a decrease in the incidence of diseased paws in the tolDC-treated group compared with the control group throughout the experiment (Fig 4A). Fig 4B summarizes the clinical scores of the animals in the three groups throughout the experimental period. tolDC transfusion significantly reduced disease severity, and the therapeutic efficacy became significant at 15 days after tolDC delivery and was sustained until the end of the experiment. This finding is supported by the changes in the appearances of the arthritic paws and the results of microCT analysis (Fig 4C–4E). To further confirm the observed phenotype, CII-specific IgG isotypes were examined in mouse sera. As shown in Fig 4C–4F, the expression levels of IgG1, IgG2a, IgG2b and IgG3 were significantly decreased in the tolDC-treated group.

**Fig 5:**
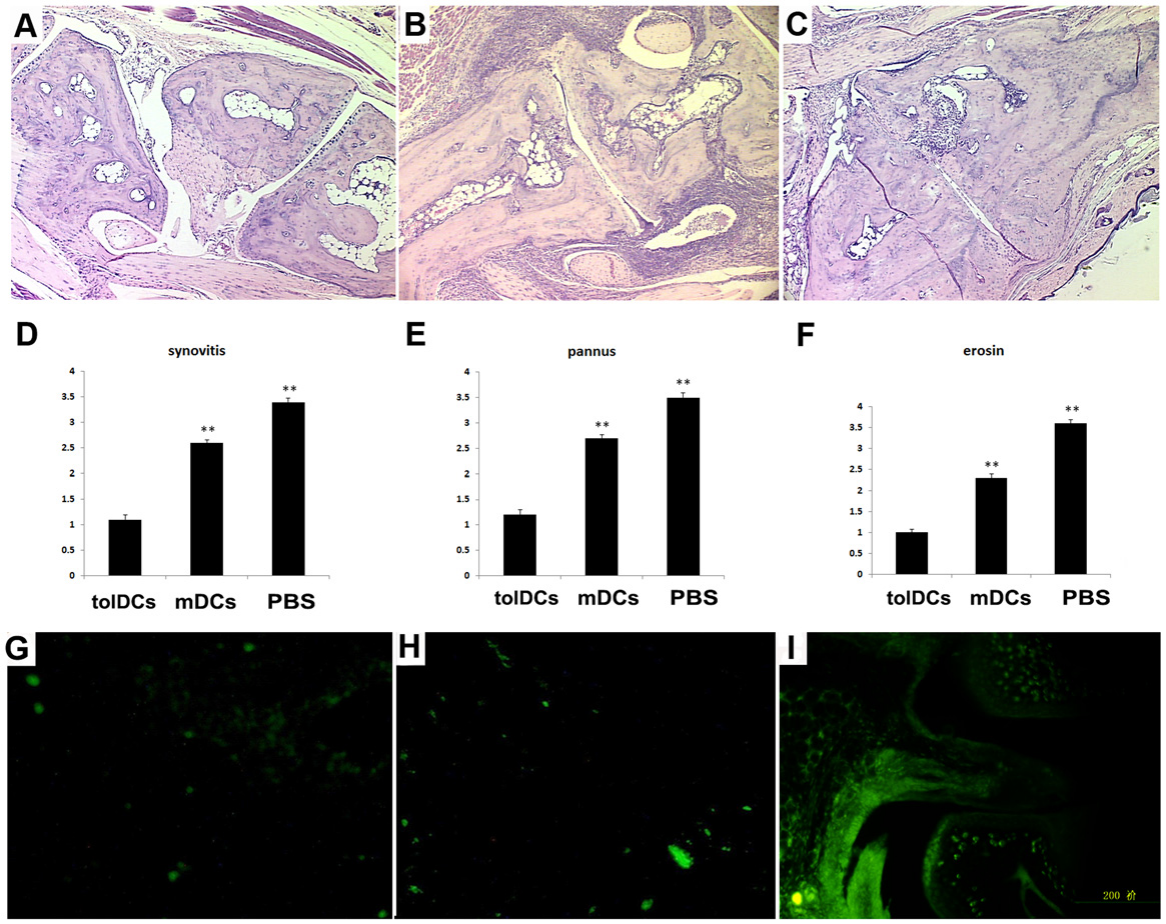
Histological examination of CIA mice. (A-C) Representative images of H&E-stained paws show different extents of damage to the joints. (D-F) Summary of the evaluation of synovitis, pannus, and erosion in all of the groups at 9 weeks after the primary immunization. (G-I) Fluorescence microscopic images of the spleen, liver and paws, respectively. *p<0.05, **p<0.01.

Histological evaluation of the arthritic paws revealed that the pathological characteristics (synovitis, pannus, and subchondral bone erosion) of the arthritic paws of the tolDCs-transfused mice were significantly alleviated compared with those of the non-treated and mDCs-transfused mice (Fig 5A–5F). Fluorescence microscopic examination revealed the presence and aggregation of GFP-labeled tolDCs in the arthritic paws (Fig 5I), particularly in the joint capsule and synovial membrane. Although sporadic fluorescent cells were identified in the spleens and livers of the recipient mice (Fig 5G–5H), no visible fluorescent cells were found in other organs or tissues, including the muscles, lungs, and brain (data not shown). Collectively, our data show that the tolDCs had targeted therapeutic effects on inflammatory arthritis *in vivo*.

### tolDCs up-regulated CD4+CD25+FoxP3+iTreg cells

**Fig 6:**
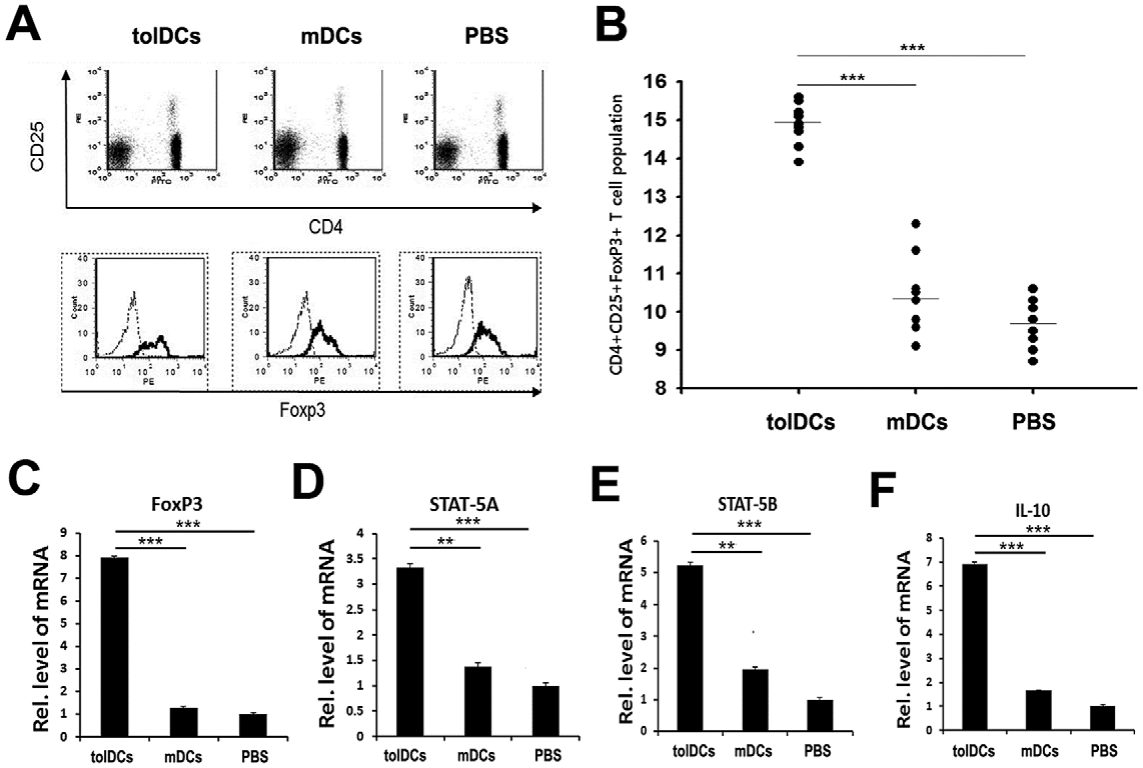
tolDCs up-regulate CD4+CD25+Foxp3+T cells. (A) Representative image of the flow cytometry plots used to identify and quantify CD4+CD25+T and CD4+CD25+Foxp3+T cells from CIA mice. (B) Statistical analysis of CD4+CD25+Foxp3+iTreg cell populations in the CIA mice in the different treatment groups. (D-F) mRNA isolated from splenocytes was evaluated by real-time PCR to determine the expression levels of the CD4+CD25+Foxp3+iTreg cell markers FoxP3, STAT-5A, STAT-5B and IL-10. ***p<0.001.

To investigate whether tolDCs ameliorate inflammatory arthritis in CIA mice by up-regulating iTreg cells, we examined the iTreg population in each group of mice. CD4+T cells, CD4+CD25+T cells, and CD4+CD25+FoxP3+T cells isolated from spleens were evaluated by tri-color staining using flow cytometry. Although the populations of CD4+CD25+T cells among the groups did not significantly differ in size (Fig 6A), the population of CD4+CD25+Foxp3+iTreg cells in the tolDC-treated group was much larger compared with those in the other two groups (Fig 6B). To confirm these results, we extracted RNA from splenocytes of the CIA mice to determine the gene expression levels of CD4+CD25+FoxP3+iTreg cell markers, including FoxP3, STAT-5A, STAT-5B and IL-10. All of the markers tested were expressed at significantly higher levels in the tolDC-treated group than in the two other groups (Fig 6C–6F). Collectively, these results suggest that tolDC transfusion dramatically increases the iTreg population, preventing the development of arthritis in CIA mice.

## Discussion

Over the past few decades, many studies have demonstrated that tolDCs function as suppressive modulators of auto-reactive T cell responses [12]. The therapeutic potential of DCs for the treatment of RA has also been well established [21–23]. RA is a common chronic autoimmune disease characterized by the infiltration of inflammatory cells and immune cells into the synovial and joint compartments. DCs have been identified as one of the major types of immune cells associated with RA, and a subgroup of DCs, referred to as tolDCs, can induce autoimmune tolerance [5]. tolDCs have received increasing attention as a promising immunotherapeutic strategy for RA. However, the use of antigen-specific tolDCs to induce immune tolerance has not been thoroughly tested. In this study, we evaluated the tolerogenic capacity of CII-pulsed tolDCs, which were propagated from mouse bone marrow in the presence of IL-10 and TGF-β. We studied the effects of these cells on the modulation of auto-reactive T cell responses *in vitro* and *in vivo*. Our results showed that tolDCs ameliorated inflammatory arthritis by up-regulating the Treg population.

A previous study by our group has established stable and functionally active tolDCs that are able to induce platelet-specific immune tolerance [24]. The current study extended our previous findings by comparing clinical-grade mDCs with tolDCs and evaluating the therapeutic effects of tolDCs on CIA. The *in vitro* experiments revealed that tolDCs secreted a large amount of IL-10 and a small amount of IL-12. Our data also suggested that these cells inhibited the proliferation of T cells, resulting in suppressed IFN-γ expression and elevated IL-10 and TGF-β production.

As expected, CII-tolDCs played a therapeutic role in ameliorating disease progression in the CIA mouse model. Several studies have shown that DCs, in combination with LPS or TNF-α, inhibit the progression of antiphospholipid syndrome [25] and type I diabetes [26, 27]. Our results support the inhibtory role of tolDCs in autoimmune diseases, although the bone marrow DCs in our study were isolated in the absence of LPS and TNF-α. Our results also demonstrate that tolDCs reduce the production of IFN-γ, which is markedly elevated in RA. Introduction of GFP-tolDCs significantly ameliorated the progression of inflammatory arthritis. GFP-CII-tolDCs predominately accumulated in inflamed joints, and almost no green fluorescence was detected in other organs *in vivo*.

With regard to cytokine production, CII-tolDC interactions resulted in decreased production of IFN-γ and increased expression of IL-10 and TGF-β (Fig 3C). High levels of expression and secretion of IL-10 and TGF-β are important for tolDC function because these two cytokines can further induce iDCs to differentiate into tolDCs, thereby maintaining tolerance to auto-antigen(s). These data indicate that CII-tolDCs have substantial tolerogenic capacities for the manipulation of memory T cells that can be applied *in vivo*. In addition, IL-10 is a well-known anti-inflammatory cytokine that has been used in clinic trials for the treatment of RA[28]. Moreover, IL-10 and TGF-β levels are decreased in RA patients compared with those of normal individuals, and their expression levels are increased in ameliorated patients, further validating our findings[29].

Dramatic increases in IL-10 and TGF-β expression as mediated by tolDCs led to the polarization of T cells into iTreg cells; thus, we analyzed the iTreg cell population in the CIA mice. The results of our *in vivo* studies suggested that tolDCs maintained their tolerogenic phenotype and function, thus promoting a potent tolerogenic microenvironment, leading to dramatic reductions in CIA symptoms and amelioration of disease progression. Regulatory T cells are central to the maintenance of self-tolerance and tissue homeostasis [30]. Our data suggested that the tolDCs caused a significant increase in the population size of CD4+CD25+FoxP3+iTreg cells in the tolDC-treated group. One possible explanation for the effect of tolDCs on disease progression in CIA mice is that they ameliorate inflammatory arthritis by up-regulating iTreg cells.

In conclusion, our data demonstrate that CII-pulsed tolDCs exhibit a stable tolerogenic phenotype and possess therapeutic potential for the treatment of inflammatory arthritis. The suppression of CIA was associated with decreased secretion of inflammatory cytokines and CII-specific antibody as well as up-regulation of the iTreg population. Our findings support the therapeutic potential of tolDCs for inflammatory arthritis and provide evidence to warrant further investigation into the design of individualized immunotherapies for RA patients.
